# Diversion colitis 25 years later: the phenomenon of the disease

**DOI:** 10.1007/s00384-017-2802-z

**Published:** 2017-03-29

**Authors:** Marek Szczepkowski, Tomasz Banasiewicz, Adam Kobus

**Affiliations:** 1 Clinical Department of General and Colorectal Surgery, Bielanski Hospital, Warsaw, Poland; 2 Department of Rehabilitation, Józef Piłsudski University of Physical Education, Warsaw, Poland; 3 Department of General, Endocrynological Surgery and Gastrointestinal Oncology, Poznan University of Medical Sciences, ul. Przybyszewskiego 49, 60–355 Poznań, Poland

**Keywords:** Diversion colitis, Rectal stump, Inflammation, Mucosal proliferation

## Abstract

**Background:**

Diversion colitis (DC) seems to be common in stoma patients, and the restoration of the continuity of the digestive tract is crucial for relief from the inflammatory process. No prospective studies of the late effects of DC on the lower gastrointestinal (GI) tract mucosa and the clinical condition of patients have been reported.

**Methods:**

Data from 23 patients who underwent stoma creation were analysed during the reversal period (A) and at an average of 3 months (B1) and 5.6 years (B2) after restoration of GI tract continuity. Every monitoring visit included endoscopy, histology and assessment of the clinical condition of patients.

**Results:**

Shortly after GI tract restoration (B1), a significant decrease in inflammation was observed. The Ki67 positivity percentage increased, but this was not significant. At an average of 5.6 years after restoration (group B2), the clinical symptoms were mild. More patients presented with endoscopically detected inflammation of the mucosa, but its severity was not significantly higher than that at 3 months after reversal. Histological inflammation was more common, and its severity was significantly higher than that shortly after reversal but similar to that before reversal. The Ki67 positivity percentage decreased at the last examination (B2).

**Conclusions:**

The results of this study show a complex recurrence of histological inflammation several years after GI tract restoration but without clinical and endoscopic inflammation and with good clinical condition. DC can potentially have a late influence on the rectal mucosa, even after stoma closure.

## Introduction

Diversion colitis was described by Glotzer et al. in 1981 [1]. Twenty-five years ago, Harig et al. published the first report of its treatment using short-chain fatty acids (SCFA) [2]. Despite the inclusion of only four patients, the study has become a classic regarding the treatment of this condition. Diversion colitis (DC) is characterized by bleeding from an inflamed large bowel mucosa that mimics idiopathic inflammatory bowel disease and that may lead to stricture formation. For many years, there were no criteria for the assessment of the severity of DC, and very little was known about the long-term effects of DC on the condition of the gastrointestinal (GI) mucosa. Some papers report the occurrence of inflammation and development of new clinical symptoms months after stoma reversal [3, 4]. To date, there are 295 publications in PubMed that include the key words ‘diversion colitis’, but in most of these reports, DC is reported incidentally during analysis of other clinical conditions.

We therefore carried out a study to examine the long-term consequences in the rectal mucosa of diversion colitis resulting from proximal stoma formation for various pathologies in a group of patients followed over 5 or more years after closure of the stoma.

## Method

The prospective study included patients who had undergone partial colonic resection with the simultaneous creation of a temporary colostomy (Table 1).

**Table 1:** Clinical characteristics of the study group

Clinical characteristics	
Age	61.6 years (min. 47; max. 81)
Sex	F 11 M 12
Primary diagnosis (reason for stoma creation)	Colorectal cancer 9 Diverticulitis 11 Bowel traumatic injury 2 Iatrogenic perforation 1
Median time of diversion (days) Specimen A	372 (min. 132; max. 982)
Median time between stoma reversal and first monitoring examination (days) Specimen B1	88 (min. 78; max. 102)
Median time between stoma reversal and second monitoring examination (days) Specimen B2	2038 (min. 1472; max. 2889)
Median length of the stump (cm)	19.5 (min. 8; max. 60)

In all cases, sigmoidostomy was created. Initially, there were 52 eligible patients. The indications for primary surgery included colorectal cancer (20 patients), diverticulitis (23 patients), volvulus of the sigmoid colon (1 patient), ovarian cancer (3 patients), traumatic bowel injury (5 patients) and iatrogenic perforation (2 patients). The initial exclusion criteria were the presence of inflammatory bowel disease such as Crohn’s disease or ulcerative colitis and anastomotic leakage after rectal cancer surgery.

**Fig. 1:**
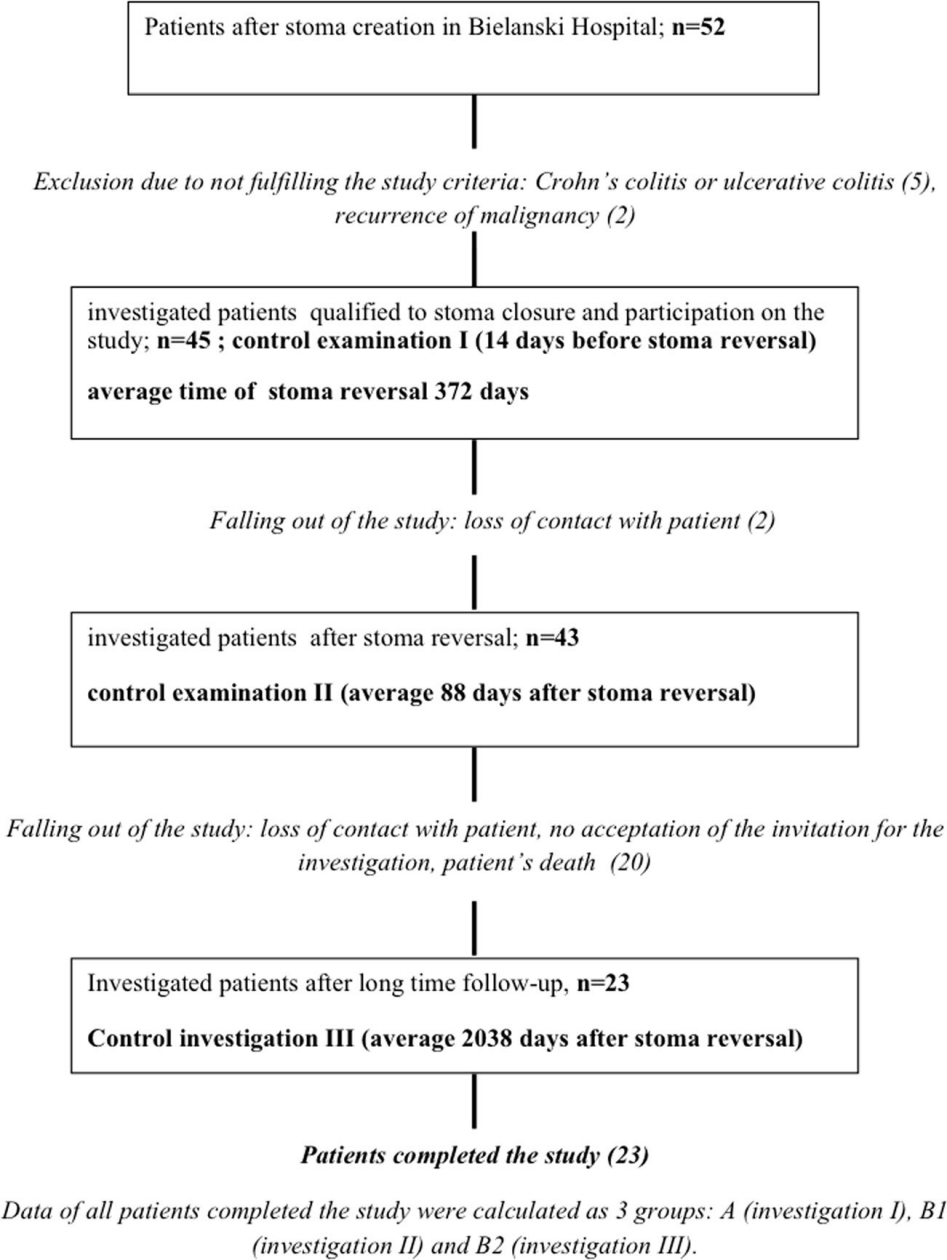
A flowchart representing the course of the study of DC patients

The 52 patients were operated on at the Bielanski Hospital between 2003 and 2008, and the continuity of their digestive tract was restored. The patients were included in a follow-up programme involving regular monitoring examinations by endoscopy, histology and clinical assessment. The long follow-up resulted in drop-out of some of the patients because of death due to other reasons, loss of contact with patient or lack of acceptation the control investigation by the well-doing patients. The final analysed group consisted of 23 patients operated on for colorectal cancer (9 patients), diverticulitis (11 patients), bowel traumatic injury (2 patients) and iatrogenic perforation (1 patient) (Fig. 1). The interval from the formation of the stoma to its closure was a median of 50.3 weeks.

### Clinical investigation

Each patient was investigated on three occasions as follows: 2 weeks before colostomy closure (period A), 3 months (period B1) after colostomy, and between 4 and 8 years (median 5.6 years) (period B2) after stoma closure. At every investigation, patients completed a questionnaire and underwent rigid rectosigmoidoscopy and mucosal biopsy using flexible colonoscopy. The basic demographic data of the patients were also recorded. The questionnaire included questions about clinical symptoms including mucous discharge, cramping abdominal pain and tenesmus. Every symptom was scored as 0—absent, 1—mild or 2—severe. To compare the results of the scoring, a control group was also created (group C). This consisted of 15 patients undergoing endoscopy before haemorrhoidectomy. No pathology other than haemorrhoids was found in these patients by colonoscopy.

### Endoscopic examination

One endoscopist performed the endoscopies for all patients. Mechanical bowel preparation before endoscopy was omitted to reduce bias. Random biopsies (three specimens) were performed on the rectum approximately 7 cm above the dentate line, even in patients with normal endoscopic findings. The endoscopist was blinded to the patient’s symptoms. The endoscopic findings were expressed according to the scoring system of Harig et al. [1] including erythema, oedema, friability, granularity and erosions. Every finding was scored as grade 0—absent, grade 1—mild or grade 2—severe.

### Histopathological examination

The histopathological severity of colitis was assessed by two independent histopathologists blinded to the patient’s clinical data. The average score of the two investigations was used in further analysis. The histopathological findings were scored as acute inflammation (0 to 1), chronic inflammation (0 to 2), eosinophilic infiltration (0 to 2), crypt architecture distortion (0 to 1), follicular lymphoid hyperplasia (0 to 1) and crypt abscess (0 to 1). The total score was validated and further defined as mild (1 to 3), moderate (4 to 6) or severe (7 to 8) [3].

### Immunohistological examination for Ki67

The monoclonal antibody Ki67 (DakoCytomation, M7240) was used to assess the cell turnover of the mucosa. In all specimens, chromogen 3,3′-diaminobenzidine (DakoCytomation, K5007) was used for antigen localization. Stained sections were analysed field-by-field at a final magnification of 400×. Each field was assigned a continuous score of positivity percentage, representative of the approximate area of immunostaining. The staining reaction obtained after the exclusion of the primary antibody was used as a negative control.

### Statistical analysis

Statistical analysis was performed using R statistical package [5]. Spearman’s rank correlation was used for ordinal data correlation analysis. Analysis of values between independent groups that did not meet the criteria for normal distribution defined by the Shapiro–Wilk test was performed using the Wilcoxon matched-pairs test for paired and the Mann–Whitney test for unpaired variables. The same tests were used when comparing ordinal values in two independent groups. When comparing two dependent groups, we used the Wilcoxon signed-rank test. Comparison of ordinal values in more than two dependent groups was performed using Friedman’s test with a Wilcoxon–Mann–Whitney test as a post hoc test. Wherever more than one analysis of the same data was performed, we used the Bonferroni correction for the *p* value.

#### Ethical considerations

The study was performed with the consent of the Ethics Committee of Centre for Postgraduate Medical Education, Warsaw, Poland.

## Results

Signs of DC were observed by endoscopy, histology and clinical score in the study group during the period of faecal diversion. Twenty-two patients showed inflammation on endoscopy, all 23 patients had histopathological inflammation and 19 showed clinical symptoms, valeted as a typical for DC (Table 2).

**Table 2:** The incidence of endoscopic, histological and clinical inflammation of the mucosa in each study group

Signs of inflammation:	Investigation A before reversal (*n* = 23)	Investigation B1 3 months after reversal (*n* = 23)	Investigation B2 5.6 years after reversal (*n* = 23)
Endoscopic inflammation (score ≥ 1) (%)	22 patients (95.6)	4 patients (17.4)	12 patients (52.2)
Histologic inflammation (score ≥ 1) (%)	23 patients (100)	22 patients (95.6)	23 patients (100)
Clinical symptoms of inflammation (score ≥ 1) (%)	19 patients (82.6)	6 patients (26.1)	6 patients (26.1)
Patients without endoscopic (0 point) histological (0 or 1 point) or clinical (0 point) signs of inflammation (%)	0 patient (0)	8 patients (34.8)	3 patients (13)

Three months after stoma closure, a significant decrease in the severity of inflammation was observed for endoscopic, histological and clinical scores (Table 3).

**Table 3:** Severity of endoscopic, histological and clinical inflammation, and Ki67 reactivity before and after stoma reversal with level of significance (Wilcoxon–Mann–Whitney test was used to determine *p* value) in study and control groups

	A	B1	B2	C	*p*
Inflammation by endoscopic score (Harig)	Mean 3.17 SD 2.14 Median 3	Mean 0.22 SD 0.52 Median 0	Mean 0.65 SD 0.88 Median 1	Mean 0.14 SD 0.36 Median 0	A vs. B1 0.0002 A vs. B2 0.0006 A vs. C 0.0000 B1 vs. B2 0.2347 B2 vs. C 0.0001
Inflammation by histological score	Mean 3.13 SD 1.82 median 3	Mean 1.48 SD 0.66 Median 1	Mean 2.44 SD 1.37 Median 2	Mean 0.5 SD 0.52 Median 0.5	A vs. B1 0.0024 A vs. B2 0.3868 A vs. C 0.0000 B1 vs. B2 0.0165
Clinical symptom score	Mean 2 SD 1.57 Median 2	Mean 0.52 SD 1.08 Median 0	Mean 0.38 SD 0.78 Median 0	Mean 0.21 SD 0.43 Median 0	A vs. B1 0.0008 A vs. B2 0.0020 A vs. C 0.0008 B1 vs. B2 1.0000 B2 vs. C 1.0000
Ki67 (% positivity)	Mean 1.05 SD 2.24 Median 0	Mean 4.28 SD 8.77 Median 0	Mean 0.69 SD 1.66 Median 0	Mean 0.24 SD 0.41 Median 0	A vs. B1 0.2327 A vs. B2 1.0000 A vs. C 0.0025 B1 vs. B2 0.0969 B2 vs. C 0.0000

There was no statistically significant change in the Ki67 positivity. At a median 5.6 years after surgery, the incidence and severity of endoscopically detected inflammation increased, but no significant difference was found between the first and second examinations after stoma reversal (B1 and B2). The incidence of histological inflammation was high, as seen in both previous examinations. The severity of inflammation increased and was significantly higher than that at B1. No significant differences were found between the severity of histological inflammation in specimens taken during diversion (A) and at B2. The Ki67 positivity percentage decreased not significantly from that at the previous examination (B1). However, the positive trend was observed.

**Fig. 2:**
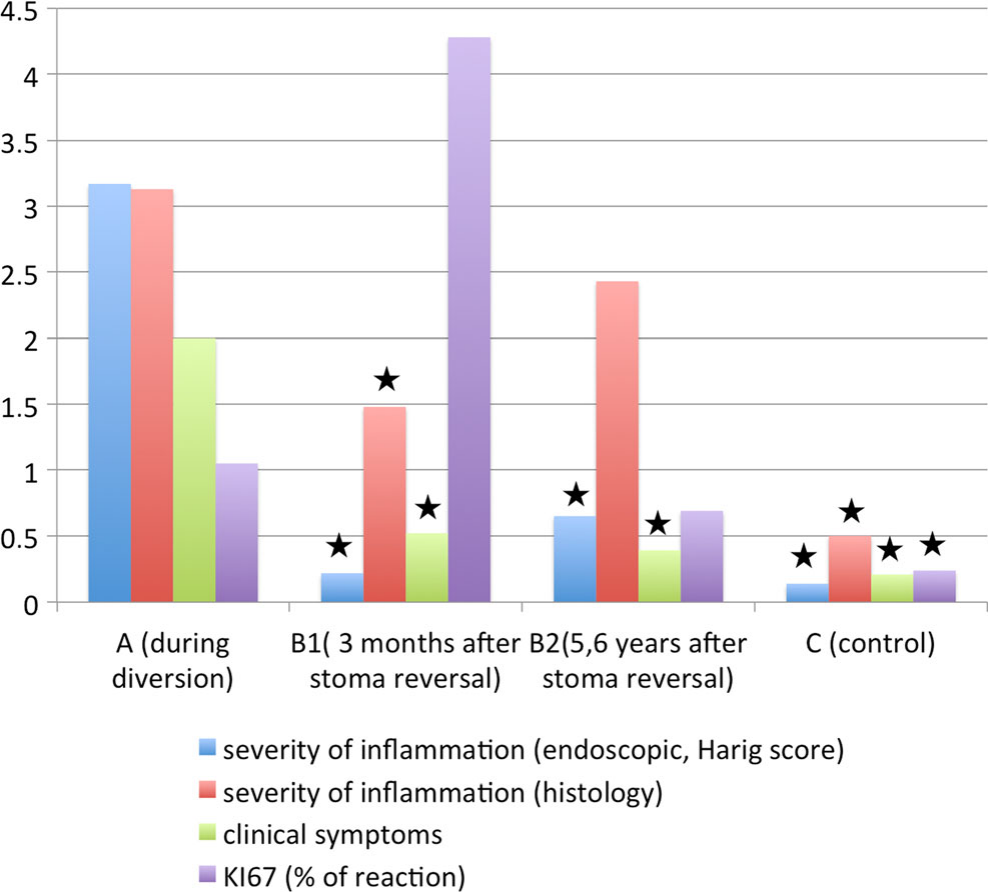
Changes in inflammation activity assessed by endoscopic, histological and clinical scores, and Ki67 positivity percentage. The statistically significant differences between analysed parameter in group A and other groups are marked with *black star* (details are in Table 3)

Changes in inflammation activity assessed by endoscopic, histopathological and clinical scores and Ki67 positivity percentage are shown in Fig. 2.

## Discussion

DC is still a problem and probably will remain so in the future [6]. However, many patients need to maintain a colostomy for long periods, and some will never attain the reconstruction of intestinal continuity. As a consequence, it is expected that DC will impair the quality of life of a significant number of patients [7].

DC, especially as identified by endoscopic and histological investigations, is very common in patients with stoma and can occur in up to 100% of these patients [4], but there is usually no significant correlation between the results of endoscopy and histology and the clinical symptoms. In our study, we found no correlation between the clinical symptoms and the endoscopic and histological severity of DC. Other authors also report no correlation between the endoscopic or histological findings and symptoms such as mucous discharge, bleeding, abdominal pain and tenesmus investigated before and after stoma reversal [3].

There are different methods of treatment for DC, of which the most effective seems to be SCFA, usually by means of butyrate enemas [8, 9]. Promising results have also been reported for autologous faecal transplantation [10]. Most authors emphasize that although DC can be treated conservatively, the best and most successful method of DC treatment is surgery to reconstruct digestive tract continuity [9, 11]. Most studies of DC are performed only up to stoma closure or for a short time thereafter, and no serious clinical problems have been reported. There is very limited follow-up information about the period after reconstruction of digestive tract continuity. In one Korean study, the incidence of symptomatic DC was reported as 63.3% after ileostomy creation, and 46.6% 5–6 months after ileostomy closure; however, 30% of patients developed diarrhoea as a new symptom [3]. Interesting data were presented by Pacheco et al. [7]: during experimental studies on the treatment of DC with either glutamine or butyrate, significant reductions in the density of collagen fibres in tissue and in the rate of apoptosis within the epithelium were observed, as well as a decrease in the levels of inflammatory cytokines. These results may suggest that in non-treated DC, both fibrosis and apoptosis may be increased.

Our observation of histological inflammation in patients investigated several years after stoma closure is a surprising result that is difficult to interpret unambiguously. However, the results of the studies presented here suggest the possibility that inflammatory lesions persist, or even in some cases, develop further, as a continuation of the morphological and molecular changes in the colonic mucosa.

A completely opposite trend was observed in the analysis of Ki67 positivity. Ki67 was used as cellular marker for proliferation, also commonly coexisting in premalignant and malignant conditions.

The low level observed during diversion increased shortly after digestive tract restoration, but after several years, the level decreased to one similar to that observed during diversion. These observed changes were not statistically significant, probably because of the high value of the standard deviation and the small numbers of patients investigated. A low level of Ki67 positivity may be correlated with low proliferation. This is supported by the observation that prolonged diversion causes involution and atrophy of the relevant segment. The clinical effect of this situation can be a poor functional outcome [12]. The low level of Ki67 positivity in DC also correlates with a low possibility of malignancy. It is also possible, comparing individuals with ulcerative colitis and those with DC, that the different tissue expression patterns exhibited by mucins may be related to a lower possibility of neoplastic transformation in gut segments that are excluded from the faecal stream because of diseases other than inflammatory intestinal diseases [13]. After stoma closure, Ki67 positivity increased (1.05 to 4.28% reactivity), while in the long-term follow-up, we observed decreasing Ki67 positivity, compared to patients with colostomy before stoma reversal (group A) and shortly after stoma closure (group B1). From the other point, it is necessary to remember that in all patients after stoma formation and reversal (A, B1, B2), positivity of Ki67 was higher than that in control group (C). It can be explained as a result of diversion colitis, stimulating the mucosal cells to proliferation; also, long time after stoma reversal, there is lack of evidences to clearly explain this fact.

The decreased level of Ki67 correlated with greater severity of morphological inflammation. Only a non-significant increase in the endoscopic signs of inflammation and no increase in the severity of clinical symptoms were observed.

The observations presented here demonstrate the long-term consequences of DC. After stoma reversal, all manifestations of colitis decreased significantly, but after several years, inflammation was observed at a morphological level. There are no data in the literature to compare with this observation, and therefore, further long-term study of DC patients is necessary. Of course, small number of patients analysed in our paper is a strong limitation of the study, and the presented results and conclusions should be interpreted with caution.

It is difficult to identify the factors potentially influencing the risk of histological inflammation several years after stoma closure. There is controversy regarding histopathological analyses of colon mucosal segments that have been excluded from the faecal stream, which demonstrates that the disease follows a variable course and that there is no characteristic pattern of changes [14–16].

A unique feature of this study is the long observation time, which sheds a slightly different light on the assessment of the natural course of DC and its consequences in the long term after the restoration of GI tract continuity. The histological inflammation of the colonic mucosa observed many years after stoma closure in our study warrants further investigation of this phenomenon.
